# Supplementary material for: Morphological plasticity of endophytic Chitinophaga pinensis
Source: Antonie Van Leeuwenhoek. 2026 Apr 11;119(5):95. doi: 10.1007/s10482-026-02300-2 (PMC13070061; doi:10.1007/s10482-026-02300-2)
Supplement: Supplementary file 1 — Supplementary file1 (DOCX 2320 KB) [file 10482_2026_2300_MOESM1_ESM.docx]

Supplementary Figures


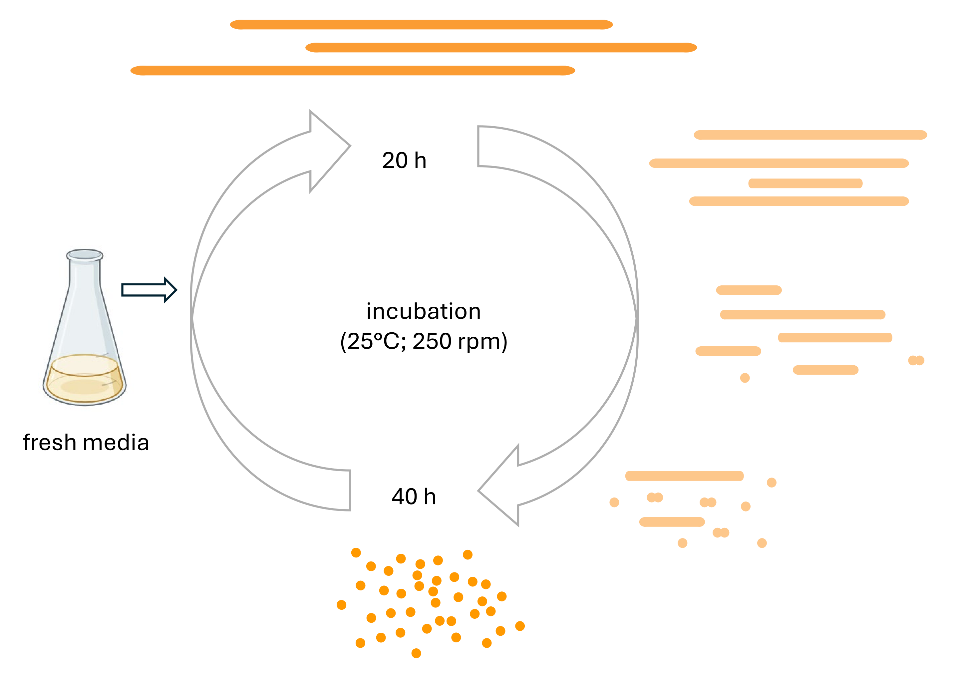


Online Resource 1. Schematic representation of the reversible morphological cycle of *C. pinensis* during growth in liquid culture (25 °C, 250 rpm). The culture transitions from predominantly spherical cells to filamentous forms within 20 h and returns to spherical morphology after 40 h. Intermediate stages are shown in lighter colour to reflect the unresolved timing of the transition. Attempts to synchronize the cell cycle and capture these intermediate forms were unsuccessful, preventing accurate determination of timing and further analysis.


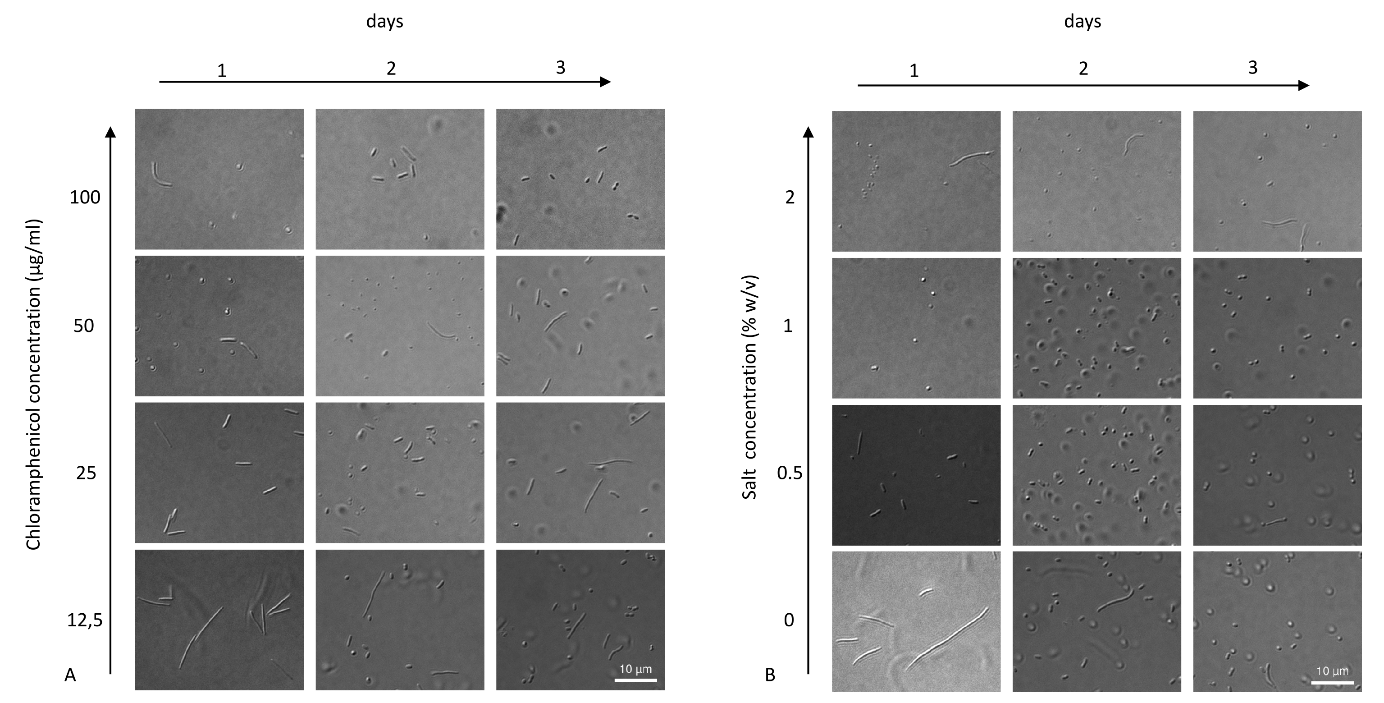


Online Resource 2. Stress reaction test of *C. pinensis* (N= 3) in 0.1x TSB with increasing chloramphenicol (µg/ml) and salt concentration (% w/v). To investigate the extent to which the formation of small spherical cells is a stress response of *C. pinensis*, cells were first harvested at the long filamentous cell stage after 20 hours of incubation and then exposed to chloramphenicol concentrations ranging from 12.5 µg/ml to 100 µg/ml (A). Chloramphenicol is an antibiotic that binds to the 50 ribosomal subunit and thus impairs protein biosynthesis. In addition, the cells were exposed to salt concentrations of 0.5% to 2% (B), which disrupt the integrity of the cell wall. A culture in 0.1x TSB without additional salt was used as a reference sample (B). Cells were imaged every 20 h for 3 days. Scale bar 10 µm.


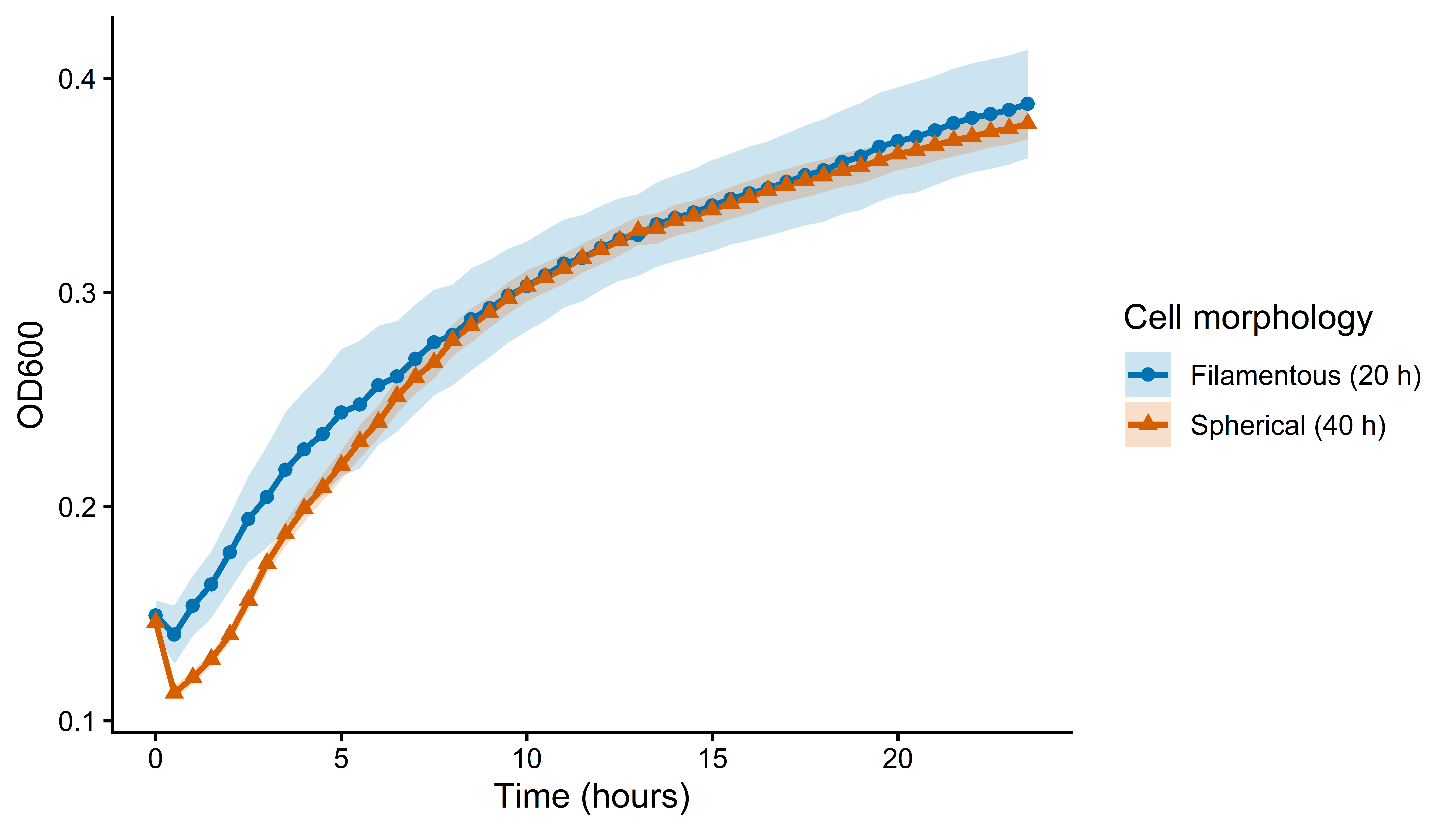


Online Resource 3. Descriptive growth curves of *C. pinensis* filamentous (harvested after 20h) and spherical cells (harvested after 40 h) following reinoculation into fresh medium at identical starting OD_600_ values. OD_600_ over time is shown as mean values ± standard deviation calculated from control wells (n = 18 per morphology).


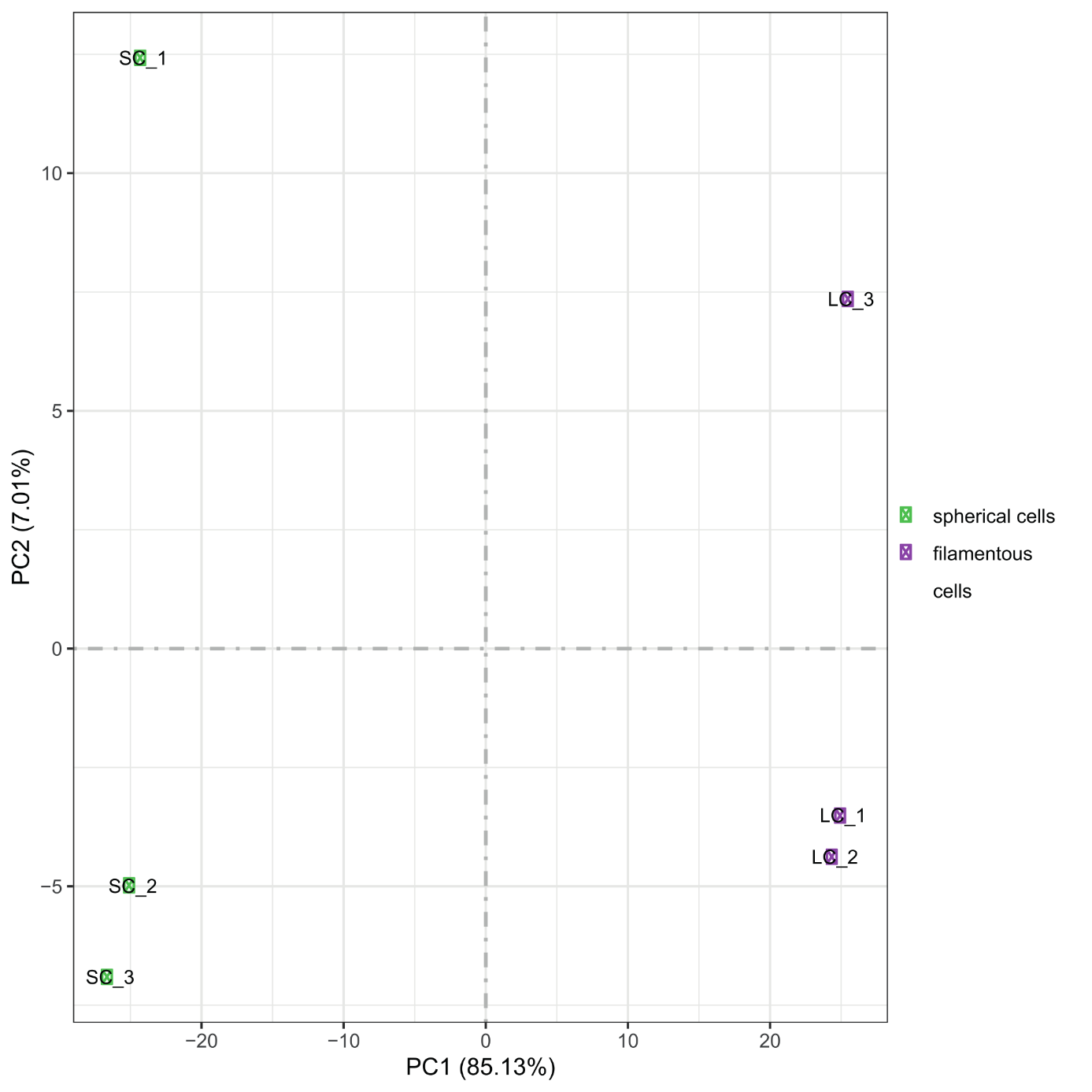


Online Resource 4. Principal component analysis (PCA) of transcriptomic profiles from *C. pinensis* cell morphologies. Samples cluster according to morphology, with PC1 explaining 85.13% of variance, separating spherical cell (SC) from filamentous cells (LC), indicating distinct transcriptional states.


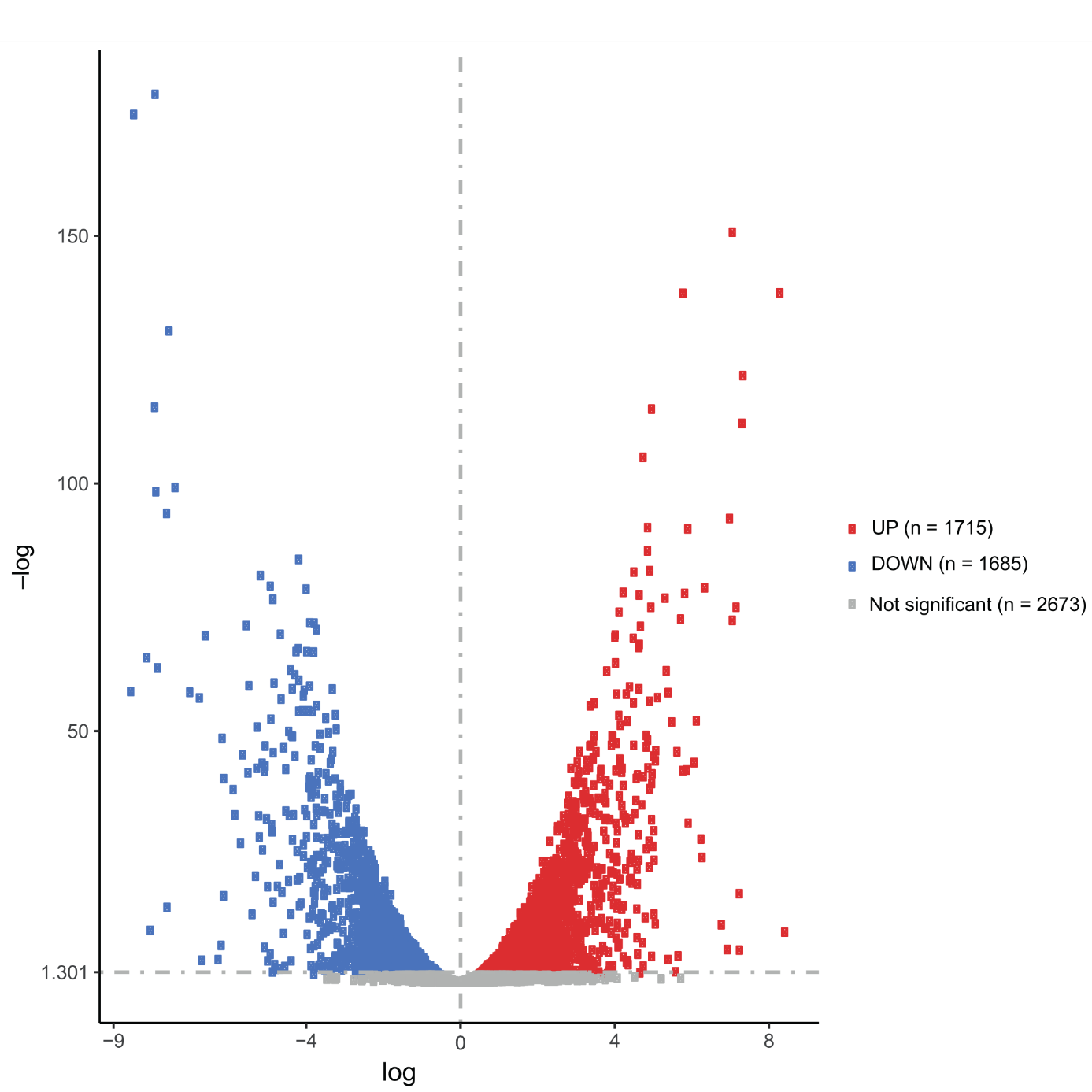


Online Resource 5. Volcano plot of differential gene expression (DEG) between spherical and filamentous *C. pinensis* cells. Genes with a fold change of 2 (FC ≥ 2) and an adjusted *p*-value (padj) of 0.05 are considered significantly regulated: red = upregulated (n = 1715), green = downregulated (n = 1685), blue = non-significant (n = 2673).


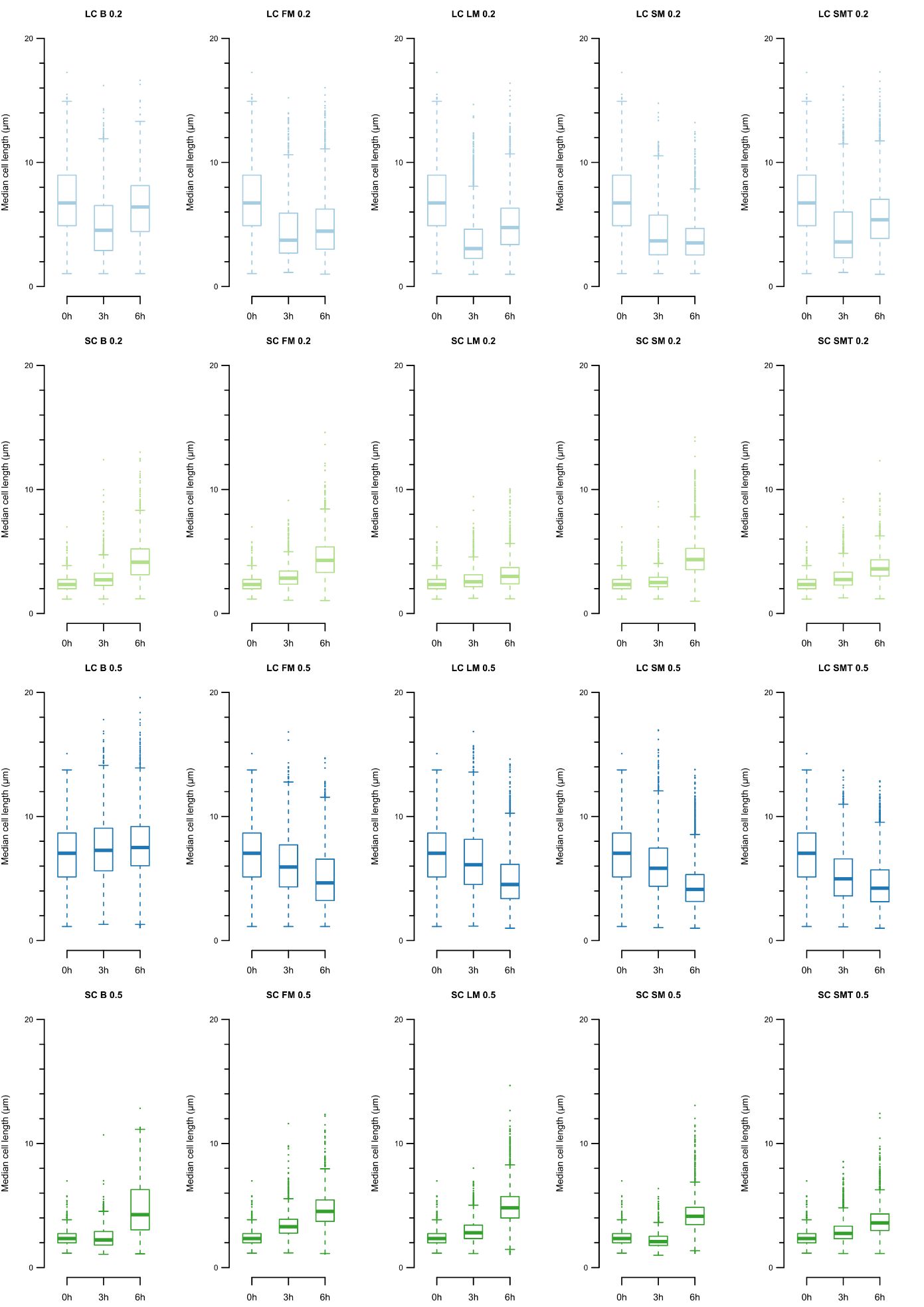


Online Resource 6. Comparison of *C. pinensis* morphological change over time under different growth conditions. (A - E; K - O) Change from an initial long filamentous cell shape (LC) with an initial cell density of OD_600_ = 0.2 (A-E) and 0.5 (K - O). (F – J; P – T) As well the transition from a small spherical cell shape (SC) with an initial cell density of OD_600_ = 0.2 (F – J) and 0.5 (P – T). Following conditions were used: buffer (B); fresh medium (FM); sterile filtrated supernatant of 20 h growth medium (LM); sterile filtrated supernatant of 40 h growth medium (SM) and sterile filtrated supernatant of 40 h growth medium supplemented with full medium (SMT). Cell lengths (µm) were measured after 3 and 6 hours after inoculation. Confidence interval 95% and N=3.

**
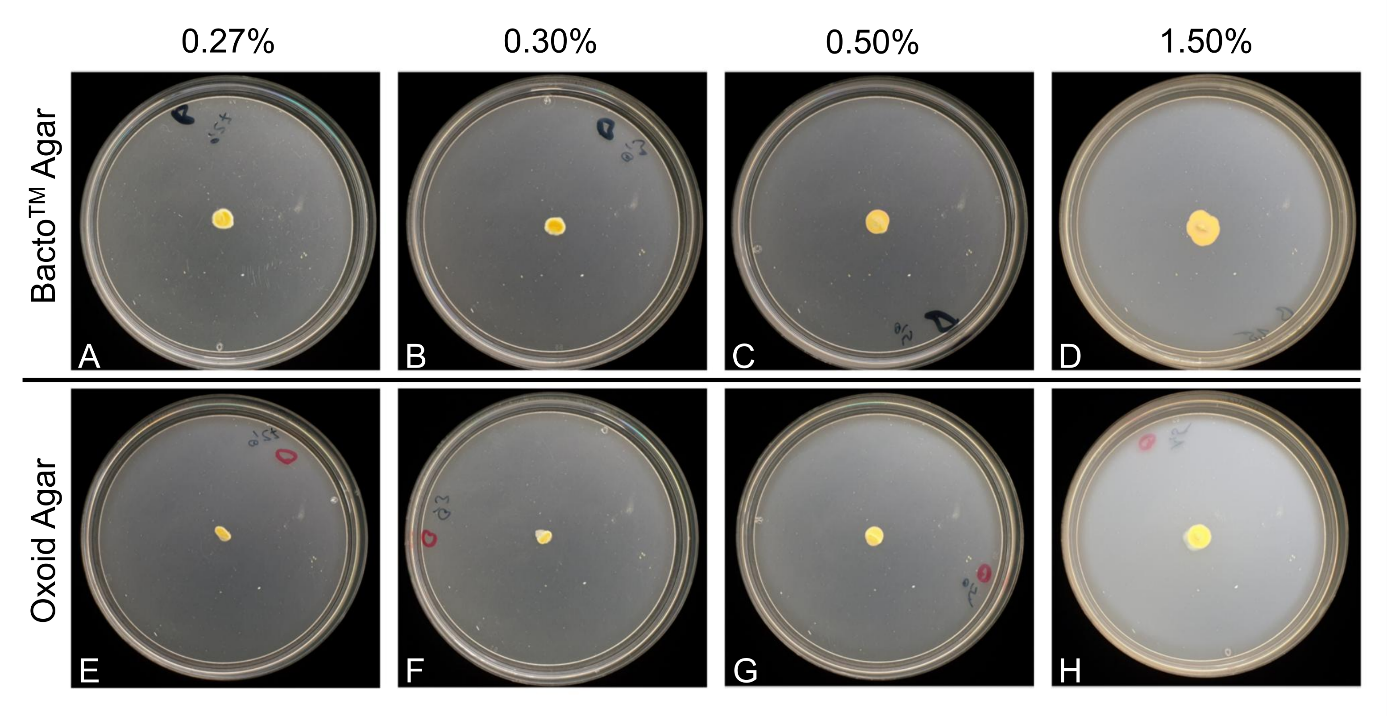
**

**Online Resource 7.** Motility assay of *C. pinensis* on 0.1x TSB media plates with increasing agar concentration ranging from 0.27% to 1.50% (w/v). Agar concentrations of 0.27–0.30% were used for swimming assays while concentrations ≥0.5% were used for swarming assays. (A–D) Plates prepared with Bacto^TM^ Agar and (E–H) plates prepared with Oxoid LP0011 as agar source. Under all conditions tested, *C. pinensis* remained at the point of inoculation and did not display swimming or swarming motility.


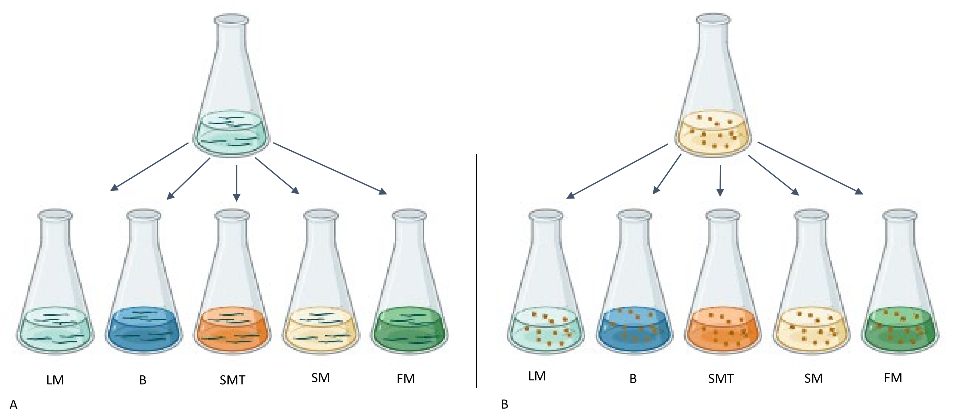


Online Resource 8. Experimental setup of the trigger assay. Long filamentous cells (LC) of a *C. pinensis* culture after 20 h incubation (25 °C; 250 rpm) and small spherical cells (SC) after 40 h of incubation in 0.1x TSB were harvested. Following respective supernatant of the 20 h (LM) and the 40 h (SM) culture was sterile filtrated and used in the setup, whereby one part of the SM supernatant was supplemented with full TSB media in a 1:10 dilution (SMT). Phosphate-buffered saline (B) and fresh 0.1x TSB medium (FM) were included as reference medium. The initial cell density for both cell morphologies was setup to an OD_600_ of 0.2 and at a higher OD_600_ of 0.5. The experiment was conducted in triplicates.

**Supplementary Methods**

Physical stressor assay

To measure the differences in viability of the filamentous and spherical cell morphologies when exposed to physical and chemical stresses, we first harvested the cells from liquid culture (50 ml) by centrifugation for 10 min. (filamentous cells) and 30 min. (spherical cells) at 8,000x g and at 4 °C. All samples were adjusted to an OD_600_ of 0.2 and stored on ice until the various treatments outlined below. Subsequently, 5 µl of each sample was drop plated in triplicates on 0.1x TSA plates (1.5% agar) and incubated at 25 °C. The plates were imaged on a light box after 2 days.

Treatment with ultrasonic

For this treatment, 1 ml of each sample was transferred to a 15 ml Falcon tube and treated in an ultrasonic bath (ultrasonic cleaner; CDS-100; AC 220 240 V 50/60 HZ; 42,000 hz) for 2 or 5 min. and then placed back on ice.

Heat treatment (60 °C)

1 ml of each sample was transferred to a Falcon tube (15 ml) and placed in a 60 °C pre-warmed water bath (Type: W 200; 220 V; 5.5 A; 50 Hz; 1200 W; Memmert GmbH + Co KG) for 2 or 5 min. and then placed back on ice.

Treatment with ultraviolet light (UV)

UV-light treatment of each sample was performed as described before (Sudo and Dworkin 1969) in a biosafety cabinet (BSC-700II-I; HMC Europe) with a UV-light source (ZW15S19W-Z436; Cnlight; Shelley). From each sample, 5 ml of the culture was transferred to a sterile petri dish (Ø 9 cm) and placed without a lid at a distance of 50 cm from the UV-light source. The exposure time was 5 or 10 min., and after treatment the samples were cooled at room temperature before plating.

Desiccation test

Filamentous and spherical cells were plated on 0.1x TSA and incubated at 25 °C for 8 weeks until the agar had completely dried out. Subsequently, 1 ml of 0.1x TSB and a sterile loop was used to scrape the cells from dried agar plates. From the resulting cell suspension, 100 µl was used to inoculate and spread onto new 0.1x TSA media plates. The plates were incubated at 25 °C and imaged after 2 weeks.

Chemical stressor assay

The chemical stressor assay shown in Figure 1B was performed as follows. Experiments to test the resistance behaviour of the two cell morphologies were carried out on 96-well plates and with biological and technical triplicates. The cells were harvested by centrifugation (30 min, 8000 g, 4 °C) and the OD_600_ of each sample was set to 0.5 – 0.6. Each well was prefilled with 180 µl 0.1x TSB and 20 µl of the respective cell suspension was added. The measurement was performed in an automated Spark® multimode microplate reader (Tecan, Switzerland) with the following parameters: temperature 25 °C; measuring time 22 - 24 h; amplitude 2 mm; frequency 810 rpm; duration 10 s; linear duration 5 s; wavelength 600 nm; 10 flashes; settle time 5 ms and an interval of 30 minutes.

Pondus Hydrogenii (pH) test

The pH test was carried out with 0.1x TSB medium (pH 7.2), which was adjusted to pH 5 and pH 8 with HCl and NaOH. Untreated 0.1x TSB (pH 7.2) was used as a positive and growth control.

Sodium dodecyl sulfate (SDS)

Sterile filtrated SDS (dissolved in 0.1x TSB) was added to the medium (0.1x TSB) at a final concentration of 0.02%; 0.2%; and 2% (w/v).

Ethanol test

Ethanol was added to the medium (0.1x TSB) at a final concentration of 0.02%; 0.2%; and 2% (w/v).

Growth rate analysis

For the evaluation and quantification of the growth parameters under the different chemical stress and control conditions, the doubling time, growth rate and carrying capacity were computed in R (version 4.3.2) using the packages growthcurver (version: 0.3.1) and lme4 (version 1.1-35.1) and the RStudio IDE. Logistic growth curves were fitted using the function SummarizeGrowth. A Welch t test was applied to compare growth parameters between the 20 h (filamentous) and 40 h (spherical) controls. Because OD_600_ values can be influenced by differences in cell morphology, OD-based growth curves analysis was used only to compare relative growth dynamics under identical measurement conditions rather than to infer biomass values.

Additionally, control samples from the chemical stressor assay (without added stressors) for each cell morphology (n = 18 per morphology) were used to generate the descriptive growth curves shown in Online Resource 3. For this purpose, OD_600_ over time was plotted as mean ± standard deviation across replicate control wells.

Stress-reaction test

Cultures of *C. pinensis* (25 °C; 250 rpm; 20 h; OD_600_ = 0.1) were centrifuged (1500x g; 30 min; 4 °C) to obtain cell pellets, which were resuspended in the following media:

Liquid media with different chloramphenicol concentrations were prepared with a chloramphenicol stock solution (25 mg/ml) and diluted with 0.1x TSB to final concentration of 100, 50, 25 and 12.5 µg/ml chloramphenicol. Media with different NaCl concentrations (0.5 – 2%) were prepared from a 10% (w/v) stock solution in 0.1x TSB. In addition, 0.1x TSB was used as the medium for the oxygen stress test. Subsequently, 1 ml of each sample was transferred to a 96-DeepWell^TM^ plate and incubated (25 °C; 250 rpm; 3 days). To prevent evaporation of the medium, the plates were sealed with a sealing film, whereby the wells of the oxygen stress test were sealed with 3 layers of sealing film. To prevent additional oxygen from entering the oxygen stress test, the sealing film was only cut open for one row of wells for each examination. Every 20 h, 10 µl was taken from each sample and the cell morphology was examined using a light microscope (Axio Imager M2; Zeiss). All tests were performed in triplicates.

Transcriptomics and data analysis

*C. pinensis* was cultured in 50 ml 0.1x TSB (25 °C; 250 rpm), and cells were harvested by centrifugation (1500x g; 15 min; 4 °C) after 20 h (filamentous cells; OD_600_: 0.16 ± 0.01) and 40 h (spherical cells; OD_600_: 0.9 ± 0.1) of growth. Before RNA extraction, the cells were briefly inspected by differential interference contrast (DIC) microscopy, which confirmed the expected dominant morphology (filamentous at 20 h and spherical at 40 h). However, we cannot exclude the presence of rare cells of the alternate morphology below detection. Due to the low density, the cell pellets of 3x 50 ml *C. pinensis* 20 h culture were pooled to obtain a sufficient number of cells for RNA extraction. From the 40 h *C. pinensis* culture, 10 ml were used for cell harvesting. The cell pellets were resuspended in 1 ml RNAprotect®, incubated for 5 minutes at room temperature (RT) and then washed with 0.1x TSB (centrifugation: 5000x g; 10 min; RT). Total RNA was extracted and purified using the RNeasy® Mini Kit (Qiagen; Hilden; Germany) according to the manufacturer's instructions. The removal of rRNA, library preparation, Illumina sequencing and subsequent quality control of the data were performed by Novogene Europe (Cambridge, UK). The RNA-sequence data was analysed using a standardized pipeline. The raw data was processed to remove adapter sequences and low-quality reads. The sequences were then aligned to the reference genome *C. pinensis* (accession no. LR632929 from the study [7]) annotated by Prokka using Bowtie2. Gene expression levels were quantified using FeatureCounts and differential expression analysis was performed using DESeq2 (1.20.0). Genes with an adjusted *p*-value below 0.05, as determined by DESeq2, were considered differentially expressed. Gene Ontology and KEGG pathway enrichment analyses were performed with clusterProfiler (v3.8.1).

Expression of green fluorescent protein (GFP)

Native *gap* promoter was amplified from genomic DNA of *C. pinensis* using primer pair Pgap_chi_F (5’- AGGGAATTCCGGACCGGTACCCCACAGGTCGCCCATAAATAA-3’) and Pgap_chi_R (5’- TTCTTCTCCTTTACTCATTTTACACTGTAGTTTGTAGATGAAAAATTGAG-3’), gene gfpmut3 was amplified from synthesized DNA fragment harbouring gfpmut3 (Chen *et al*. 2007) using primers gfpmut3_F (5’- ACAGTGTAAAATGAGTAAAGGAGAAGAACTTTTCACT-3’) and Chitin_GFP_R (5’- TGCATGCCTGCAGGTCGACTCTAGATATTTGTCCTACTCAGGAGAGCGTTC-3’), the aforementioned PCR products were purified and cloned into KpnI and XbaI digested pCP23 (Agarwal *et al*. 1997) via Gibson assembly. The generated construct pGWS1802, which expresses GFPmut3 under the control of the *gap* promoter, was introduced into *C. pinensis* by electroporation as described previously (Chen *et al*. 2007). The successful introduction of pGWS1802 was validated by colony PCR on transformants which were able to grow on CYE agar containing tetracycline (100 µg/mL), using primers pair gfpmut3_F and Chitin_GFP_R.
